# Follow-up of pre-motor symptoms of Parkinson’s disease in adult patients with Gaucher disease type 1 and analysis of their lysosomal enzyme profiles in the CSF

**DOI:** 10.1186/s13023-023-02875-3

**Published:** 2023-10-02

**Authors:** Matheus Vernet Machado Bressan Wilke, Fabiano Poswar, Wyllians Vendramini Borelli, Kristiane Michelin Tirelli, Dévora Natalia Randon, Franciele Fátima Lopes, Fernanda Bender Pasetto, Fernanda Medeiros Sebastião, Gabrielle Dineck Iop, Larissa Faqueti, Layzon Antonio da Silva, Francyne Kubaski, Artur Francisco Schumacher Schuh, Roberto Giugliani, Ida Vanessa Doederlein Schwartz

**Affiliations:** 1 Medical Genetics Service, Hospital de Clínicas de Porto Alegre (HCPA), Ramiro Barcelos St., 2350, 3Rd Floor, Porto Alegre, RS 90035-007 Brazil; 2 Postgraduate Program in Medical Sciences, Universidade Federal do Rio Grande do Sul (UFRGS), Porto Alegre, RS Brazil; 3 Postgraduate Program in Genetics and Molecular Biology, UFRGS, Porto Alegre, RS Brazil; 4 Neurology Service, Hospital de Clinicas de Porto Alegre, Porto Alegre, RS Brazil; 5 LEIM- Genetics Laboratory - Serviço de Genética Médica, Medical Genetics Service, HCPA, Porto Alegre, RS Brazil; 6 BRAIN Laboratory, HCPA, Porto Alegre, RS Brazil; 7 Biodiscovery Laboratory, HCPA, Porto Alegre, RS Brazil; 8 Biochemical Genetics Laboratory, Greenwood Genetics Center, Greenwood, SC USA; 9 Department of Pharmacology, UFRGS, Porto Alegre, RS Brazil; 10 Department of Genetics, UFRGS, Porto Alegre, RS Brazil; 11 Pharmacology and Therapeutics research program, UFRGS, Porto Alegre, Brazil

**Keywords:** Gaucher disease, Parkinson`s disease, Non-motor symptoms, Cerebrospinal fluid

## Abstract

**Background:**

Parkinson’s disease (PD) is the second most common neurodegenerative disease worldwide. Its classic motor symptoms may be preceded by non-motor symptoms (NMS). Population studies have identified *GBA* variants as risk factors for idiopathic PD. The increased risk of PD has also been suggested in other Lysosomal Storage Disorders (LSDs). Objective: To assess the evolution of the prevalence of NMS compatible with PD in a cohort of South Brazilian adult patients with Gaucher Disease (GD) type 1, already evaluated 3 years ago (2018). Cerebrospinal Fluid (CSF) was collected to assess the levels of LSD enzymes (beta-hexosaminidases, beta-glucuronidase) and biomarker of macrophage activation (chitotriosidase, ChT), compared to controls (metachromatic leukodystrophy, MLD). Cognition was evaluated by the Montreal Cognitive Assessment (MoCA) questionnaire, daytime sleepiness by the Epworth Sleepiness Scale (ESS), depression by Beck´s Inventory, constipation by the Unified Multiple System Atrophy Rating Scale (UMSARS) scale, and REM sleep behavior disorder by the single-question screen. Hyposmia was assessed with Sniffin’ Sticks (SST).

**Results:**

Nineteen patients completed the follow-up (mean age of the sample was 44 years; range, 26–71). The patient with the highest number of NMS at the baseline (4 including the lowest SST score) was diagnosed with PD four years later. Apart from an improvement in the ESS score, no other statistical significance was found between the number of NMS between the first and second evaluation, nor between patients with one L444P variant (n = 5) and the rest of the cohort. CSF was collected in five patients (mean age of the sample was 40 years, range 30–53. A significant difference was found in the mean CSF activity levels of beta-hexosaminidases and beta-glucuronidase between GD1 and MLD patients. Mean ChT (CSF) was 62 nmol/h/mL in GD patients and 142 in MLD (n = 6) patients.

**Conclusions:**

The patient with the highest number of NMS in our 2018 cohort was the one that developed PD, corroborating with the importance of this longitudinal follow-up. CSF and plasma analysis might allow a better understanding of the neurodegenerative processes connecting PD and the lysosomal environment. Further analysis is needed to understand this relationship.

## Background

Gaucher disease (GD, OMIM #230800) is an inborn error of metabolism caused by a deficiency of the lysosomal enzyme beta-glucocerebrosidase (GCase), which is encoded by the gene *GBA*. The prevalence of GD ranges from 0.70 to 1.75 per 100,000 individuals [1]. GD is conventionally classified into three clinical forms based on neurological involvement. Type 1 (GD1) is considered non-neuronopathic, whereas types 2 and 3 are considered neuronopathic forms [2]. According to the Human Gene Mutation Database (HGMD) database, more than 690 damaging variants in the *GBA* gene have been described, with c.1226A > G (N370S) being the most frequent in the GD type 1 population.

After its first association in 1996, pathogenic variants in *GBA* are now considered the most commonly known genetic risk factor for Parkinson’s disease (PD) [3]. PD patients with GD-associated variants (PD-*GBA*) appear to have a different disease progression, developing dementia and psychosis significantly earlier than those without pathogenic variants [4]. Although GD patients carry two pathogenic variants in *GBA*, there is no significant difference in PD risk between homozygous and heterozygous carriers of *GBA* variants, as was previously believed [5]. The motor symptoms of PD can be preceded by a prodromal period of up to 20 years. The so-called non-motor symptoms (NMS) that occur during this prodrome, such as hyposmia, rapid eye movement (REM) sleep disorder, daytime drowsiness, constipation, depression, and anxiety, may represent the beginning of the pathological process of PD and appear to be more often found in PD-*GBA* [6, 7].

The accumulation of α-synuclein (α-syn), a hallmark of PD, has been reported to be closely related to the endolysosomal pathway [8]. Moreover, other lysosomal enzymes have been suggested to play a role in PD pathogenesis, as exemplified by many lysosomal disorders presenting parkinsonism as a symptom [9]. Within this context, our main objective was to evaluate the evolution of NMS of PD in a cohort of Brazilian patients with GD1 who were already evaluated in a previous study [10]. We also aimed to assess the cerebrospinal fluid (CSF) activity of other lysosomal enzymes (alfa-mannosidase, beta-glucuronidase, beta-hexosaminidase) and chitotriosidase (ChT) in GD1 patients to better understand their role in disease pathogenesis.

## Methods

### Non-motor symptoms assessment

**Fig. 1:**
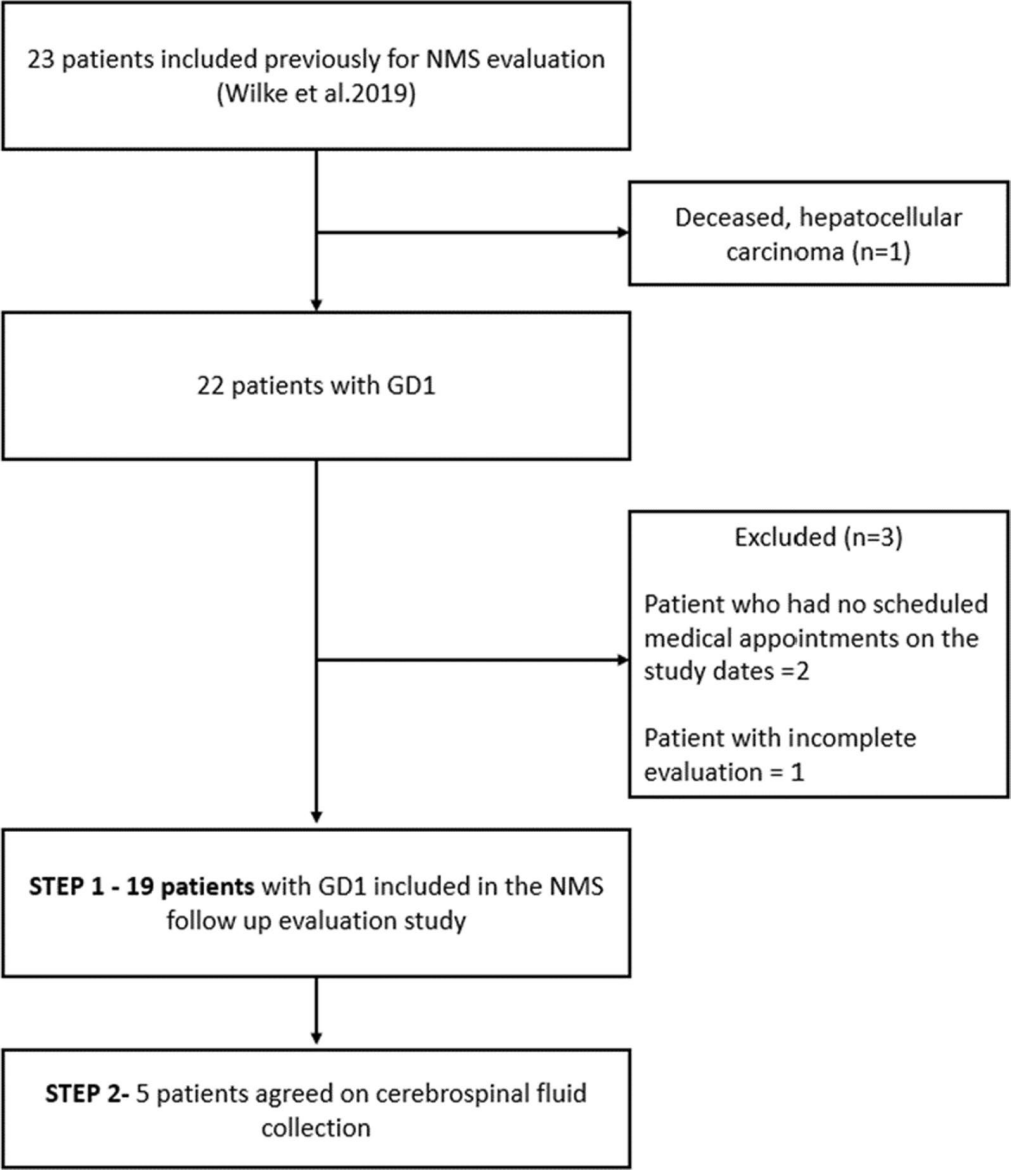
Flow diagram of patient enrollment

This is an observational, cross-sectional study with a convenience sampling. GD type 1 patients seen at the Reference Center for GD in Rio Grande do Sul, Brazil, that were part of the 2018 NMS assessment were invited to participate. The enrollment happened during their routine follow-up visits from November/21 to July/22. Figure 1 shows a flow diagram of patient enrollment. After signing a research consent form the patients were evaluated by two clinical geneticists (MW and FP) who collected the clinical data (including the self-report questionnaires to evaluate NMS of PD) and assessed for the presence of parkinsonian manifestations. Motor symptoms of PD were assessed with part III of the Unified Parkinson’s Disease Rating Scale (MDS-UPDRS III). Cognition, daytime sleepiness, depression, constipation, and REM sleep behavior disorder were evaluated respectively by the Montreal Cognitive assessment (MoCa, cutoff for cognitive impairment < 26), the Epworth Sleepiness Scale (ESS, cutoff point > 10), the Beck Depression Inventory (BDI) (cutoff for depression > 14), the subscale of the Unified Multiple System Atrophy Rating Scale (UMSARS, cutoff for constipation ≥ 2) and the validated Single-Question Screen for REM sleep behavior disorder (RBD-1Q) [29]. Hyposmia was assessed with the 12-item Sniffin’ Sticks smell identification test (SST, cut off for hyposmia < 9/12). All procedures were evaluated and scored identically at follow-up to those performed at baseline.

### Lumbar puncture

Lumbar puncture was performed after written informed consent. The procedure was performed by a trained physician in clinical neurology (WVB). Lumbar puncture was performed before the patient’s routine medical appointment between 11:00 and 1 pm. The samples were collected in sterile polypropylene tubes. Care was taken so that none of the samples was contaminated by blood during the procedure. The samples were centrifuged for 10 min at 4000 g, and 0.5-mL aliquots were immediately frozen at − 80 °C.

### Lysosomal activity (CSF and Plasma)

Analysis of the activity of beta-glucuronidase, alpha-mannosidase, beta-hexosaminidase (HEX) and chitotriosidase (Cht) were assessed in cerebrospinal fluid (CSF) and plasma from patients with GD type 1. Lysosomal enzyme activity is measured at acidic pH in plasma, leukocytes, fibroblasts, amniocytes, chorionic villi and cerebrospinal fluid. An artificial fluorogenic substrate is used, which is degraded by the enzyme and releases the fluorescent fraction (methylumbelliferone). This activity is measured against a methylumbelliferone standard curve, and the activity is expressed in nmol/h/mL (plasma, CSF) and in nmol/h/mg protein (specific activity for the other samples). Metachromatic leukodystrophy (MLD) patients were used as controls due to sample availability.

### Statistical analysis

Numerical variables with normal distribution were expressed as means and standard deviations. On the entire sample, Wilcoxon signed-rank test was performed for the variables MoCa, BSI, ESS, UMSARS, RBD-1Q, SSI, and total NMS number. Effect size was calculated with Rank-Biserial correlation with its respective confidence interval of 95% at both baseline and follow-up. In addition, the same analysis was performed dividing the sample between GD1 patients with at least one L444P (alternate nomenclature of the variant p. Leu483Pro; NM_000157.3; in *GBA*) variant. The difference between lysosomal enzymes in the CSF were calculated using the Mann–Whitney test. Their respective effect sizes and 95% confidence intervals were calculated for the Rank-Biserial correlation. All analyzes were performed in JASP (JASP Team, 2022) version 0.16.3.0.

## Results

Nineteen patients completed the follow-up. The mean age of the sample was 44 years (range, 26–71) with a mean time of treatment duration of 15 years (range, 6–28). Most patients (*n* = 18/19) had at least one N370S allele, with the most common genotype being N370S/Rec*Nci*I (*n* = 6/19). Five patients presented one allele with the L444P pathogenic variant. The complete demographic and clinical information can be found in Table 1. The only NMS in the cohort that showed a significant difference from baseline was ESS that decreased after 3 years (mean = 9.7 SD = 4.5 at baseline and mean = 7.36 SD = 5.0 at follow-up). ESS was also the most frequent NMS found in our cohort in both baseline and follow-up. There was no difference between the patients that were heterozygous for one L444P (n = 5) variant regarding the overall number of NMS or their scores compared to the rest of the cohort. At the follow-up, eight patients had at least two NMS (mean age = 52 years) from which two (P9, P18) had an increase in the NMS total number, three (P4, P17, P19) had a decrease and two (P10, P15) remained with the same number of NMS. The complete demographic and clinical information can be found in Table 2.

**Table 1:** Demographic and clinical characteristics of patients with Gaucher disease type 1 (n = 19)

Patient	Gender	Age (years)	Genotype	Treatment duration (years)	Current treatment	Platelets (× 10^9^/L)	Hb (g/dL)	Lyso-Gb1 (nmol/L)	ChT activity (nmol/hr./mL)	Severity scores	
										SSI	DS3
1	M	26	N370S/G202R	18	ERT	171	14.9	234.1	4951	0	0.66
2	F	27	N370S/L444P	6	ERT	225	12.5	127.0	3095	1	0
3	F	29	N370S / L444P	13	ERT	166	11.5	61.6	425	1	0
4	M	30	N370S/IVS9 + 1G > A	19	ERT	149	15.4	453.0	1988	3	1.25
5	F	31	N370S/*RecNciI*	22	SRT	146	11.6	371.0	3699	0	2.5
6	F	32	N370S/L461P + IVS10 + 1G > T	28	ERT	162	12.9	NA	7044	3	1.06
7	M	35	N370S/Rec*Nci*I	21	ERT	212	16.1	77.0	1243	3	1
8	F	37	N370S/L444P	19	ERT	182	13.3	192.7	2306	0	0
9	F	43	N370S/ *RecNciI*	7	ERT	221	13.0	589.0	736	2	1
10	M	42	N370S/L444R	11	ERT	142	14.4	112.0	1194	0	0
11	F	44	N370S/L444R	14	ERT	171	15.0	122.0	3022	1	0.66
12	M	46	N370S/L444P	17	ERT	290	13.2	NA	2495	4	4.7
13	M	49	N370S / L444P	10	ERT	199	14.4	15.0	116	5	0.4
14	F	53	E349K/S366N	10	ERT	285	14.7	23.6	643	3	1.8
15	F	55	N370S/Rec*Nci*I	20	ERT	405	14.7	113.0	1216	5.66	13
16	M	61	N370S/Rec*Nci*I	10	ERT	160	16.1	64.6	1170	3	0
17	F	66	N370S/*RecNciI*	24	ERT	153	13.8	70.0	581	22	2.4
18	M	68	N370S/N370S	12	ERT	160	14.2	85.8	1529	0	0.5
19	F	71	N370S/L444R	11	ERT	309	13.9	120.6	257	10	4.5

*ERT* enzyme replacement therapy. *SRT* substrate reduction therapy. *SSI* Zimran Severity Score Index (mild = 0–10; moderate = 11–19; severe ≥ 20). *DS3* Disease Severity Score (mild =  < 3.00; moderate = 3.00–5.99; marked = 6.00–19). *Hb* hemoglobin. *Plat* platelet count*. lyso-Gb1* glucosylsphingosine (normal range: < 12 nmol/L) *ChT* chitotriosidase activity (normal range: < 78.5 nmol/hr./mL). *NA* not available

**Table 2:** Evolution of the scores of the scales used to evaluate non-motor symptoms of Parkinson’s disease in patients with Gaucher disease type 1 at baseline (pre) and after a 3 year follow-up (post) (n = 19)

Patient	L444P +	MoCa pre	MoCa post	BDI pre	BDI post	*ESS* pre	*ESS* post	UMSARTS pre	UMSARTS post	RBD-1Q pre	RBD-1Q post	SST pre	SST post	Total NMS pre	Total NMS post
1*	N	30	30	4	7	6	0	0	0	N	N	10	12	0	0
2	Y	26	**25**	**16**	0	**11**	6	1	1	N	N	10	11	2	1
3	Y	29	28	5	0	8	4	1	1	N	N	12	11	0	0
4*	N	**23**	26	3	8	**17**	**15**	0	0	**Y**	**Y**	11	9	3	2
5	N	26	27	4	5	9	4	1	0	N	N	11	10	0	0
6	N	26	28	3	9	**11**	7	1	1	N	N	10	10	1	0
7	N	26	28	2	NP	8	9	1	0	**Y**	**Y**	9	11	1	1
8*	Y	27	27	12	4	**11**	**11**	1	1	N	N	11	10	1	1
9*	N	26	29	6	9	**13**	**11**	1	0	N	**Y**	12	12	1	2
10	N	26	26	0	0	**18**	**18**	0	0	**Y**	**Y**	12	9	2	2
11	N	30	29	7	2	3	4	1	1	N	N	12	11	0	0
12	Y	29	28	2	7	10	**13**	0	0	N	N	10	10	0	1
13	Y	29	NP	4	7	**15**	9	0	0	N	N	12	12	0	0
14*	N	**22**	**25**	**25**	29	1	3	**2**	**2**	N	N	10	10	3	3
15*	N	**22**	**22**	**33**	6	7	**11**	0	0	N	N	11	11	2	2
16	N	26	26	14	10	**12**	8	0	1	N	N	**6**	9	2	1
17*	N	**22**	NP	**17**	18	4	0	**2**	**2**	N	N	**4**	**5**	4	3
18*	N	26	**24**	3	0	7	0	0	0	N	**Y**	11	9	0	2
19*	N	19	**17**	4	6	**13**	7	**2**	0	**Y**	N	9	9	4	3
Mean		25.8	26.2	8.6	7.0	9.7	7.3	0.7	0.5			10.1	10	1.4	1.1
SD		3.0	3.1	8.8	7.1	4.5	5.1	0.7	0.7			2.1	1.6	1.3	1.0

Altered results are presented in bold. Asterisk denotes patients who screened positive for more than one non-motor symptom of Parkinson’s disease. BDI Beck Depression Inventory (cutoff for depression > 14), ESS Epworth Sleepiness Scale (cutoff for increased daytime sleepiness > 10), UMSARS Unified Multiple System Atrophy Rating Scale (cutoff for constipation ≥ 2), SST Sniffin’ Sticks Test (cutoff for hyposmia < 9/12), MoCa Montreal Cognitive assessment (cutoff for cognitive impairment < 26), RBD-1Q Single-Question Screen for REM Sleep Behavior Disorder (cutoff being a positive answer to the single question), NP not performed. L444P + Presence of the L444P in one of *GBA* alleles

P17 presented the highest number of NMS, being also the only patient to present hyposmia (SS = 4) at baseline when she was 62 years old. The patient started treatment with enzyme replacement therapy (ERT) at the age of 42 due to anemia, thrombocytopenia, splenomegaly, and hyposmia. No motor symptoms were present in 2018. In the 2021 follow-up she was diagnosed with PD due to bradykinesia and tremor. To rule out other causes of parkinsonism a genetic panel for genes related to PD and parkinsonism was done showing, apart from the *GBA* variants, a VUS in *VPS13C* that was not deemed relevant to the case. The patient has been showing good response to the levodopa treatment. No other patients in the cohort showed signs of parkinsonism.

**Fig. 2:**
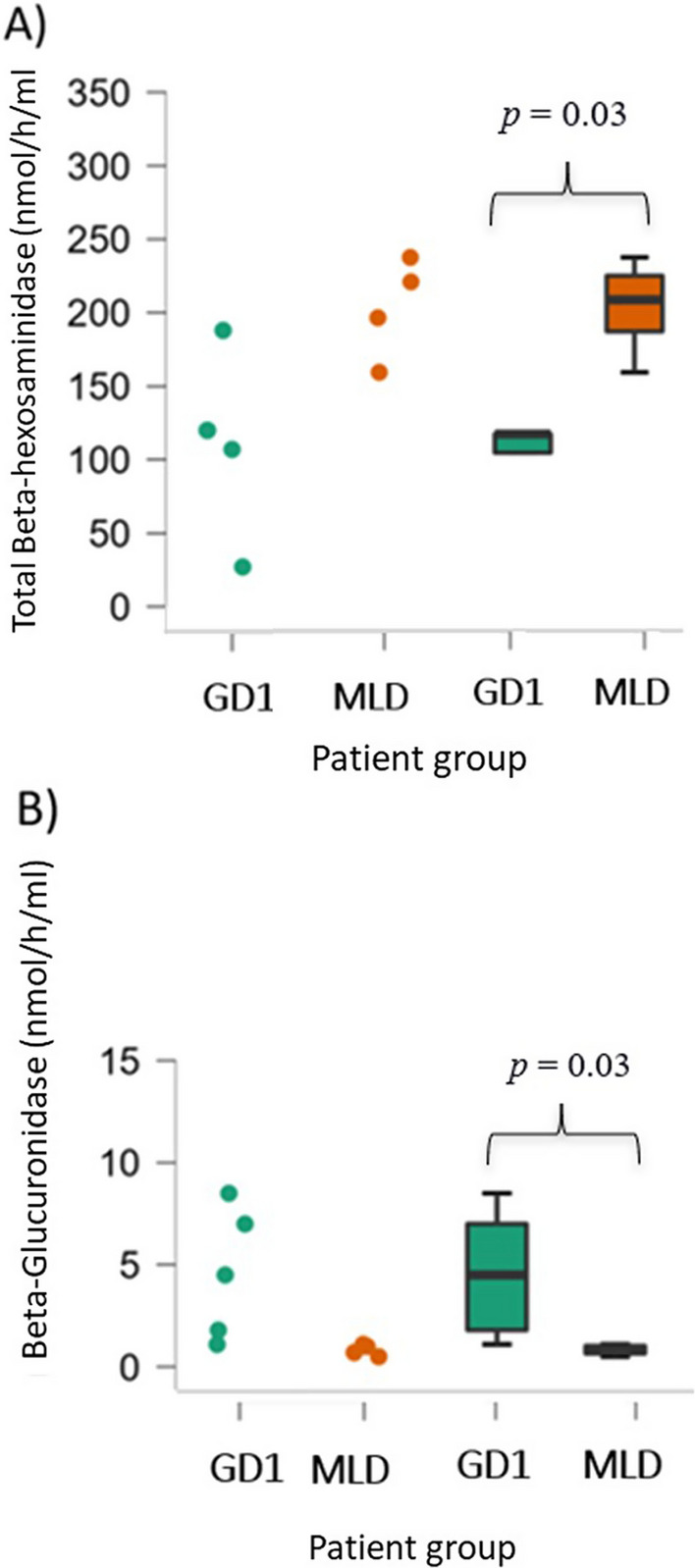
Lysosomal enzymes activity in the cerebrospinal fluid of patients with Gaucher Disease type 1 (GD1, n=5) and Metachromatic leukodystrophy (MLD, n=6)

Five patients (P4, P7, P8, P11, P14) had their CSF analyzed for lysosomal biomarkers (mean age of the sample was 40 years, range 30–53) and six patients with MLD were used as controls. Regarding the CSF biomarkers, significant difference (p = 0.03) was found between the groups for HEX activity, which mean value was 112.4 ± 57.3 nmol/h/mL in the GD group versus 208.2 ± 34.7 nmol/h/mL observed in the MLD group. Same behavior (p = 0.03) was observed for beta-glucuronidase, with mean values of 4.6 ± 34.7 nmol/h/mL and 1.1 ± 0.5 nmol/h/mL, respectively for GD and MLD groups, as illustrated in Fig. 2. Beta-hexosaminidase B was increased in the GD group with mean = 47.6 ± 53.5 nmol/h/mL (reference range on plasma = 1000–2857 nmol/h/mL with no available reference range in CSF) in comparison to the MLD group, which mean value was 20.7 ± 8.7 nmol/h/mL. The analysis of these enzymes on plasma of GD were normal (values not shown). ChT levels exhibited a higher increase in the MLD group (mean = 142.8 ± 101.2 nmol/h/mL; reference range on plasma = 8.8–132 nmol/h/mL with no available reference range in CSF) in comparison to the GD group (mean = 70 ± 51 nmol/h/mL) even though they were not statistically different- (p=0.08). The complete biomarker assessment in CSF can be found in Table 3.

**Table 3:** Descriptive statistics for lysosomal enzymes measured in groups 1 (Gaucher Disease Patients) and Group 2 (Metachromatic Leukodystrophy Patients) (Reference range for enzymes are not available in cerebrospinal fluid)

Enzyme	Group	N	Mean	SD	*p*
Beta-hexosaminidase A	1	5	94.8	70.0	
	2	4	187.5	32.2	0.063
Beta-hexosaminidase B	1	5	43.4	41.3	
	2	4	20.7	8.7	0.556
Total Beta-hexosaminidase	1	5	112.4	57.3	
	2	4	208.2	34.7	0.037
% Beta-hexosaminidase A	1	5	56.0	49.1	
	2	4	90.0	4.2	0.268
Beta-glucuronidase	1	5	4.6	3.2	
	2	6	1.1	0.5	0.035
Chitotriosidase	1	5	62.0	43.7	
	2	6	142.9	101.3	0.082
Alpha-Mannosidase	1	5	1.9	1.7	
	2	6	1.27	0.5	0.713

## Discussion

PD has been increasingly associated with the lysosome after the discovery of several autosomal dominant and recessive genes associated with PD correlated with other genes important for the functioning of this organelle. The aggregation of α-syn has been reported in the brain of patients and animal models of different LSDs, including GD, Fabry disease, Niemann-Pick disease, Sandhoff disease, Tay-Sachs disease, MLD, β-galactosialidosis and GM2 gangliosidosis, Krabbe’s disease, and Neuronal Ceroid Lipofuscinosis [9].

Elevated ChT activity has already been described in other neurological disorders including stroke, multiple sclerosis and cerebral adrenoleukodystrophy (C-ALD) [11]. ChT levels even correlated with the degree of severity of white matter lesions which corroborates to the importance of macrophages and astrocytes in the natural immune response of these demyelinative lesions [11, 12]. The more severe involvement of the brain in MLD might be the responsible for the higher levels of ChT found in this group in comparison to the GD patients. Higher levels of Cht in CSF were also demonstrated in other LSDs with more severe neurological involvement than GD1as GM1-gangliosidosis and GM2-gangliosidosis, for example [13]. Another possible explanation for this finding is that the GD group is treated with ERT which is already extensively associated with the lowering of ChT levels [14]. However, as this treatment doesn’t cross the brain blood barrier and ChT is not clearly demonstrated to breach it, the ERT role on the lower levels of ChT in the GD group still needs to be further elucidated [15].

Lower HEX activity was found in the GD group when compared to MLD patients in our cohort. In a study that analyzed α-syn, phosphorylated tau protein and lysosomal enzymes in CSF of 71 PD patients compared with 45 neurological controls, CSF GCase activity was significantly decreased, and HEX activity was moderately increased in the PD group [16]. HEX is associated with the Sandhoff disease caused by the accumulation of ganglioside GM2 and with shared features with PD including the α-syn inclusions frequently found in the Sandhoff disease brain of both patients and mouse models [8, 17]. Interestingly, a new study that biochemically characterize α-syn changes in the transgenic HEX knockout mice model showed that HEX expression decreases α-syn association to lipid compartments and prevented nigrostriatal accumulation of AAV6-overexpressed α-syn [17]. To the best of our knowledge this is the first time that low HEX is described in the CSF of GD patients. We hypothesize that a lower HEX activity in GD might also be contributing to the increased risk of PD found in these patients; however, a bigger sample of patients is necessary to establish a better association.

We hypothesize that the usefulness of CSF endolysosomal enzyme activity levels as biomarkers of PD, and their role in the prodromal period of PD, are likely to be of greater value when used in combination with measures of other substrates, such as glycosphingolipids and complex gangliosides. We could not find any explanation in the literature for the increase of beta-glucuronidase seen in the GD group, as this enzyme was mostly associated with bacterial meningitis [18]. Currently, there is no treatment available that can stop the progression of PD. However, new studies suggest that re-establishing normal levels of GCase, which has been reported to be reduced in PD patients, may be a treatment option for patients carrying *GBA* variants, as demonstrated by some animal models [17, 19]. One limitation of our study was the availability of few lysosomal enzymatic assays and the restricted number of GD CSF samples, which did not allow for a proper assessment of the lysosomal environment in GD patients as intended.

The NMS assessment allows for a more complete follow-up of GD patients, as it was recently reported that most GD patients would like to be informed about their PD risk in the clinical setting by the physician in charge at the time of GD diagnosis [20]. After a three-year follow-up, it was interesting to note that the patient with the highest number of NMS and the lowest SST score was the one who developed PD after two years (P17). In a study that performed a six-year longitudinal evaluation of 31 GD1 patients, the GD subjects had significantly worse scores in UPSIT (Pennsylvania Smell Identification Test), UMSARS (Unified Multiple System Atrophy Rating Scale), MoCA, and MDS-UPDRS III compared to controls, with hyposmia being a predictive factor for deterioration in this population. Due to the importance of NMS in our cohort, we advocate for the routine assessment of smell, even by directed anamnesis, as multiple tools can be used in clinical practice to assess hyposmia [22].

In another two-year follow-up study, it was shown that RBD and UPDRS III scores were significantly worse in GD patients and PD-*GBA* variants compared to controls at baseline [23]. On the other hand, olfactory and cognitive assessments were lower in the GD group and did not differ significantly from baseline [23]. Although we followed up our patients for almost the same period, no consistent pattern was observed between both cohorts. One hypothesis for the lack of difference in our NMS follow-up compared to Avenali's and Beavan's cohorts might have been the older mean age of these cohorts (mean ages = 52 and 61 years, respectively) compared to ours [21, 23].

It was intriguing to observe that ESS significantly improved, and the BDI score was not worsened during the follow-up, which happened during the SARS-CoV-2 pandemic, as these were described as common during this period [24, 25]. We hypothesize that the changes in habits during the pandemic, mainly working habits (including less time spent in commute due to working from home), might be responsible for these findings.

The term "variant severity" has been used in the literature to define the effect of the variant on the phenotype of patients with GD, classifying them as "severe" (e.g., 84 GG, V394L, D409H, L444P) or "mild" (e.g., N370S, R496H) [26, 27]. However, this terminology has been criticized because the pathogenic variant N370S, which is considered "mild," has great phenotypic heterogeneity. Homozygotes can remain asymptomatic for decades, while others can present severe clinical manifestations in childhood, including hepatosplenomegaly and pancytopenia [28]. It has been reported that motor, cognitive, olfactory, and psychiatric symptoms are more severe in PD carriers of "severe" *GBA* variants compared to those carrying mild or no *GBA* variants [26]. We could not find any difference in the NMS assessment between patients presenting at least one L444P allele and the rest of the cohort. Our single patient presenting motor symptoms (P17) only had "mild" variants, which leads to the hypothesis that other factors influence the neurodegeneration of PD beyond the patient's genotype.

## Conclusions

Hyposmia was the NMS that best predicted the development of PD in our cohort, showing important prognostic value. We recommend a detailed neurological physical examination focusing on PD motor symptoms starting at the age of 30 in GD patients. HEX activity was lower in the CSF of patients when compared to the MLD group. More studies will be necessary to assess whether this could also be a contributing factor to the increased risk of PD in these patients. A combination of biomarkers, both clinical and in CSF, might help define the optimal time to intervene with neuroprotective therapy for PD when it becomes available.

## Data Availability

If necessary, further information is available from the corresponding author on reasonable request. Further data sharing is not applicable to this article as no datasets were generated or analyzed during the current study.

## Abbreviations

α-syn: α-Synuclein
ChT: Chitotriosidase
CSF: Cerebrospinal fluid
ERT: Enzyme replacement therapy
ESS: Epworth Sleepiness Scale
GCase: Beta-glucocerebrosidase
GD: Gaucher disease
GD1: Gaucher disease type 1
HEX: Beta-hexosaminidase
HGMD: Human gene mutation database
LSD: Lysosomal storage disorders
MLD: Metachromatic leukodystrophy.
NMS: Non-motor symptoms
PD: Parkinson`s disease
PD-*GBA*: PD patients with GD-associated variants
REM: Rapid eye movement
SST: Sniffin’ sticks
